# Association between serum 25-hydroxyvitamin D levels and bilateral vestibulopathy: A case-control study

**DOI:** 10.1016/j.clinsp.2026.101114

**Published:** 2026-09-09

**Authors:** Dongxiao Zhou, Junjie Guo, Xiaoqiu Liang, Junyu Wu, Zhihui Zheng, Haiwei Huang, Weiwei Qi, Zhezhi Deng

**Affiliations:** Department of Neurology, The First Affiliated Hospital, Sun Yat-sen University, Guangdong Provincial Key Laboratory of Diagnosis and Treatment of Major Neurological Diseases; National Key Clinical Department and Key Discipline of Neurology, China

**Keywords:** 25-hydroxyvitamin D, Bilateral vestibulopathy, Vitamin D deficiency, Vestibular disorders

## Abstract

**Background:**

The etiology of Bilateral Vestibulopathy (BVP) is complex. Given the close association between vitamin D and various vestibular disorders, including benign paroxysmal positional vertigo and vestibular neuritis, this study aims to investigate the relationship between serum vitamin D levels and BVP.

**Methods:**

This single-center retrospective case-control study included 35 patients with BVP and 70 age- and sex-matched healthy controls (HC). Demographic information, clinical data, including 25-Hydroxyvitamin D (25(OH)D) levels, and vestibular assessment results were collected.

**Results:**

Serum 25(OH)D levels were significantly lower in the BVP group than in the HC group (18.58±5.80 vs. 24.25±5.91 ng/mL, p < 0.001). Insufficient or deficient vitamin D levels were observed in 97.14% of patients in the BVP group (deficiency, 60.00%; insufficiency, 37.14%). No significant association was found between vitamin D insufficiency or deficiency and the disease duration, season of visit, residential latitude, or underlying medical conditions in patients with BVP. Binary logistic regression analysis showed that serum 25(OH)D levels were associated with BVP, with an odds ratio of 0.846 (95% CI 0.765–0.935; p = 0.001).

**Conclusion:**

Serum 25(OH)D levels were significantly lower in patients with BVP than in HC, suggesting an association between vitamin D insufficiency/deficiency and BVP.

## Highlights

• First study on serum vitamin D levels and Bilateral Vestibulopathy (BVP).
• Serum 25(OH)D levels were lower in BVP patients than in healthy controls.
• Vitamin D insufficiency/deficiency was independently associated with BVP.

## Introduction

Bilateral Vestibulopathy (BVP) is a chronic vestibular syndrome characterized by physical instability when walking or standing, and blurred vision when moving around. These symptoms are exacerbated in the dark and on uneven surfaces.^1^ Patients with BVP have an increased risk of falling, which severely affects their daily mobility.^2^ Ototoxicity, bilateral Meniere's disease, and intracranial infections are considered definite causes of BVP. However, the etiology, pathomechanisms, and susceptibility of some patients remain unclear.^3^

Vitamin D is the main hormone that regulates the balance between calcium and phosphorus, as well as bone metabolism. It is primarily obtained through diet or by converting the cholesterol precursor 7-dehydrocholesterol after exposure to ultraviolet B radiation. Vitamin D insufficiency or deficiency is common.^4^^,^^5^ While vitamin D is best known for its role in the skeletal system, recent studies have found strong associations between vitamin D deficiency and cardiovascular diseases, diabetes, and autoimmune diseases. The active form of vitamin D is 1,25-Dihydroxyvitamin D (1,25(OH)_2_D). However, its precursor, 25-hydroxyvitamin D (25(OH)D), is the indicator that is routinely tested in clinical practice as it has a longer half-life and reflects vitamin D storage levels. It is involved in the pathophysiological processes of several diseases, potentially by regulating the immune system, inhibiting the production of pro-inflammatory factors, and reducing oxidative stress.^6^^,^^7^

Low levels of vitamin D have been linked to the development of several neurological disorders. Vitamin D also nourishes the peripheral nervous system, thereby promoting myelinogenesis, neuronal differentiation, and axonal homogeneity.^8^^,^^9^ In patients with vestibular disorders, low vitamin D levels may increase the risk of Benign Paroxysmal Positional Vertigo (BPPV) and Meniere's disease, and vitamin D supplementation may reduce the recurrence rate of these conditions.^10^, ^11^, ^12^ Wu et al. found that vitamin D deficiency may increase the risk of Vestibular Neuritis (VN), and suggested that this may be due to its anti-inflammatory and immunomodulatory effects.^13^ However, the association between low vitamin D levels and bilateral vestibulopathy remains unclear. In this study, we examined serum 25(OH)D levels in patients with BVP and analyzed the association between vitamin D levels and clinical characteristics, as well as findings from vestibular evaluations.

## Materials and methods

### Patients

This single-center retrospective case-control study included 35 patients with BVP who were admitted to the Department of Neurology at our hospital between 2020 and 2025. Based on a sample size calculation, 70 age- and sex-matched Healthy Controls (HC) were selected. The researchers made the diagnoses based on the criteria for BVP developed by the consensus document of the Classification Committee of the Bárány Society, and grouped patients according to their diagnoses and vestibular assessment results. Patients with comorbid psychiatric disorders, cognitive impairment, external ophthalmoplegia, or any other conditions that could affect vestibular function testing, were excluded. Information was collected on the participants' clinical profiles, as well as their disease duration, latitude of residence, season of visit, current health status, smoking, and alcohol consumption.

### Measurement of serum 25(OH)D levels

Serum 25(OH)D levels were measured in all patients using an electrochemiluminescence immunoassay (25-Hydroxyvitamin D assay kit; Roche Diagnostics GmbH, Germany) on a Cobas 6000 analyzer. All assays were performed according to the manufacturer’s instructions and under the standard internal quality-control procedures of the clinical laboratory. Serum calcium, total cholesterol, high-density lipoprotein cholesterol, low-density lipoprotein cholesterol, triglycerides, C-Reactive Protein (CRP), and Erythrocyte Sedimentation Rate (ESR) were also tested, with all blood samples collected in the fasting state early in the morning. According to the Endocrine Society Clinical Practice Guideline of America, serum vitamin D levels of <20 ng/mL are categorized as deficient, 20–30 ng/mL as insufficient, and greater than 30 ng/mL as normal.^14^

### Assessment of vestibulo-ocular reflex (VOR) function in patients with BVP

The VOR function was tested in all patients using a video head impulse test (Interacoustics, EyeSeeCam, Denmark) and caloric testing (Interacoustics, VisualEyesTM525, Denmark). We considered a pathological horizontal angular VOR gain of < 0.6 on both sides, or a reduced caloric response (sum of both thermally induced peak SPVs on each side < 6°/s), to be a positive test result. Bilateral horizontal angular VOR gain and caloric response (sum of the maximal peak velocities of slow-phase nystagmus on both sides) were recorded.

### Statistical analyses

Statistical analyses of all data were performed using IBM SPSS Statistics 22. One-way analysis of variance was used to analyze data that conformed to a normal distribution, and the results were expressed as the mean ± standard deviation. Non-normally distributed measurements were compared using the Kruskal-Wallis H test, with the results described in terms of medians and interquartile ranges. Categorical variables were compared and presented as frequencies and percentages using either the Chi-Squared test or Fisher's exact test. Pearson's and Spearman's tests were used to perform correlation analyses between vitamin D levels and other parameters. Finally, a binary logistic regression model was used to analyze the factors independently associated with BVP. A p-value <0.05 was considered to be statistically significant.

## Results

### Demographic and clinical data of patients with BVP, VN, and HC

Table 1 presents detailed basic information and clinical data for both groups. It is clear from the table that there are no significant differences between the groups in terms of age (BVP, 61.14±12.39; HC, 59.69±9.39; p = 0.503), sex (male:female = 17:18 vs. 35:35; p = 0.890), season of visit (p = 0.260), and latitude of residence (p = 0.905). The presence of hypertension or diabetes, smoking, alcohol consumption, serum calcium levels, lipid levels, CRP levels, and ESR was not significantly different between the two groups.

**Table 1:** Demographic and clinical characteristics of patients.

**Variables**	**BVP (n = 35)**	**HC (n = 70)**	**p-value**
Age (years)	61.14±12.39	59.69±9.39	0.503
Sex (Male/Female)	17/18	35/35	0.890
Disease duration (months)	15 (2,48)	‒	‒
Season, n (%)			
Spring	10 (28.57)	18 (25.71)	0.260
Summer	9 (25.71)	22 (31.43)	‒
Fall	14 (40.00)	18 (25.71)	‒
Winter	2 (5.71)	12 (17.14)	‒
Latitude (°)	23.13 (22.33, 23.39)	23.13 (23.02, 23.13)	0.905
Smoking, n (%)	3 (8.57)	13 (18.57)	0.179
Alcohol abuse, n (%)	3 (8.57)	5 (7.14)	1.000
Hypertension, n (%)	13 (37.14)	25 (35.71)	0.886
Diabetes, n (%)	6 (17.14)	7 (10.00)	0.351
Serum calcium (mmol/L)	2.22±0.09	2.24±0.12	0.242
25(OH)D (ng/mL)	18.58±5.80	24.25±5.91	<0.001
Deficiency, n (%)	21 (60.00)	15 (21.43)	<0.001
Insufficiency, n (%)	13 (37.14)	46 (65.71)	<0.001
Normal, n (%)	1 (2.86)	9 (12.86)	<0.001
Total cholesterol (mmol/L	4.82±1.17	4.94±1.19	0.644
HDL-C (mmol/L)	1.06 (0.87,1.30)	1.08 (0.94, 1.39)	0.242
LDL-C (mmol/L)	3.04 (2.22, 3.67)	2.92(2.39, 3.58)	0.856
Triglycerides (mmol/L)	1.40 (1.01, 2.50)	1.17 (0.90, 1.84)	0.159
C-reactive protein (mg/L)	1.40 (0.50, 2.90)	1.00 (0.50, 3.38)	0.865
Erythrocyte sedimentation rate (mm/h)	19.00 (12.00, 33.75)	20.00 (8.00, 29.75)	0.639

BVP, Bilateral Vestibulopathy; HC, Healthy Controls; 25(OH)D, 25-hydroxyvitamin D; HDL-C, High Density Lipoprotein Cholesterol; LDL-C, Low Density Lipoprotein Cholesterol.

### Serum 25(OH)D levels between patients with BVP and HC

Insufficiency or deficiency of 25(OH)D was prevalent in both groups, including the HC group. However, this was more pronounced in patients with BVP, with 97.14% of patients having insufficient or deficient 25(OH)D levels (deficiency, 60.00%; insufficiency, 37.14%). The concentration of 25(OH)D was significantly lower in the BVP group than in the HC group (18.58±5.80 vs. 24.25±5.91 ng/mL, p < 0.001, Table 1)

### Differences in serum 25(OH)D levels among subgroups of patients with BVP

We further analyzed subgroups of patients with BVP according to sex, season of visit, smoking and drinking habits, and the presence of underlying disease. No significant effects on serum 25(OH)D levels were observed in relation to sex, season, hypertension, diabetes, smoking, or alcohol consumption in patients with BVP (Table 2).

**Table 2:** Serum 25(OH)D levels among subgroups of patients with BVP.

Variables	Mean ± SD (ng/mL)	Range (ng/mL)	p-value
Sex			
Male (n = 17)	19.40±6.17	9.00‒31.90	0.422
Female (n = 18)	17.80±5.48	8.70‒28.70	‒
Season			
Spring (n = 10)	16.71±6.03	8.70‒27.40	0.182
Summer (n = 9)	20.64±6.14	14.30‒31.90	‒
Fall (n = 14)	19.44±5.23	10.90‒28.70	‒
Winter (n = 2)	12.60±1.41	11.60‒13.60	‒
Smoking			
Smoker (n = 3)	19.73±2.47	17.00‒21.80	0.724
Non-smoker (n = 32)	18.47±6.03	8.70‒31.90	‒
Alcohol abuse			
Alcoholic (n = 3)	18.17±5.13	12.30‒21.80	0.900
Non-alcoholic (n = 32)	18.62±5.93	8.70‒31.90	‒
Hypertension			
Hypertension (n = 13)	19.67±7.01	11.20‒31.90	0.400
Non-hypertension (n = 22)	17.93±5.01	8.70‒27.40	‒
Diabetes			
Diabetes (n = 6)	20.93±5.78	11.20‒27.40	0.280
Non-diabetes (n = 29)	18.09±5.78	8.70‒31.90	‒

BVP, Bilateral Vestibulopathy; 25(OH)D, 25-hydroxyvitamin D.

### Correlation between 25(OH)D levels and other clinical parameters in patients with BVP

As shown in Table 3, 25(OH)D levels in patients with BVP are not significantly correlated with age, disease duration, latitude, serum calcium level, lipid level, CRP level, or ESR. Further correlation analysis revealed no significant association between the degree of vestibular dysfunction and vitamin D levels in patients with BVP. Specifically, there was no significant association between serum 25(OH)D levels and the mean gain in the bilateral horizontal angular VOR or caloric response in patients with BVP (*r_s_* = 0.079, p = 0.657; *r_s_* = −0.323, p = 0.063).

**Table 3:** Correlation between serum 25(OH)D levels and other parameters, as well as vestibular evaluation results.

**Variables**	***r* or *r_s_* (95% CI)**	**p-value**
Age	0.042 (−0.341 ‒ 0.408)	0.813
Disease duration	0.132 (−0.254 ‒ 0.495)	0.448
Latitude	−0.136 (−0.498 ‒ 0.231)	0.437
Serum calcium	−0.192 (−0.507 ‒ 0.165)	0.277
Total cholesterol	−0.297 (−0.584 ‒ 0.102)	0.093
HDL-C	−0.137 (−0.449 ‒ 0.180)	0.446
LDL-C	−0.252 (−0.553 ‒ 0.148)	0.157
Triglycerides	−0.133 (−0.486 ‒ 0.236)	0.460
C-reactive protein	0.007 (−0.322 ‒ 0.340)	0.968
Erythrocyte sedimentation rate	−0.142 (−0.502 ‒ 0.255)	0.438
Mean gain of VOR	0.079 (−0.321 ‒ 0.406)	0.657
Caloric response	−0.323 (−0.589 ‒ 0.029)	0.063

25(OH)D, 25-Hydroxyvitamin D; HDL-C, High Density Lipoprotein Cholesterol; LDL-C, Low Density Lipoprotein Cholesterol; VOR, Vestibulo-Ocular Reflex.

### Binary logistic regression analysis

In comparing patients with BVP and HC, collinearity diagnostics were performed for age, sex, presence of hypertension or diabetes, serum calcium, serum 25(OH)D levels, total cholesterol, CRP, and ESR (Variance Inflation Factor < 10), and multifactorial logistic regression analyses showed that sufficient 25(OH)D levels were independently associated with lower odds of BVP (Odds Ratio = 0.846; 95% Confidence Interval, 0.765–0.935, p = 0.001; Table 4). The Hosmer-Lemeshow goodness-of-fit test indicated adequate model calibration (χ² = 10.879, df = 8, p = 0.209). The C-statistic for the multivariable model was 0.744 (95% CI: 0.640–0.848), indicating acceptable discrimination. Sensitivity analyses were performed by sequentially removing each covariate from the full model. The adjusted odds ratio for serum 25(OH)D remained stable, ranging from 0.831 to 0.854, and remained statistically significant in all reduced models (all p < 0.05), indicating that the inverse association was not driven by any single confounder.

**Table 4:** Identification of risk factors for BVP using a logistic regression analysis.

**Variables**	**Odds ratio (95% CI)**	**p-value**
Age	1.009 (0.956‒1.065)	0.738
Sex	1.433 (0.512‒4.012)	0.493
Hypertension	1.236 (0.392‒3.901)	0.718
Diabetes	1.753 (0.448‒6.855)	0.420
Serum calcium	0.032 (0.000‒5.287)	0.186
Serum 25(OH)D levels	0.846 (0.765‒0.935)	0.001
Total cholesterol	0.840 (0.544‒1.296)	0.429
C-reactive protein	0.910 (0.770‒1.076)	0.269
Erythrocyte sedimentation rate	1.011 (0.984‒1.038)	0.438

BVP, Bilateral Vestibulopathy; 25(OH)D, 25-Hydroxyvitamin D.

## Discussion

Vitamin D3, which is synthesized in the skin from 7-dehydrocholesterol, as well as vitamins D3 and D2 obtained from the diet, is hydroxylated in the liver to form 25(OH)D. It is further metabolized in the kidneys to its active form, 1,25(OH)₂D, which binds to the vitamin D receptors in tissues to exert its biological effects. Humans primarily obtain vitamin D from skin exposure to sunlight, a factor influenced by season, exposure duration, and latitude. Vitamin D levels are affected by diet, medication, cholesterol metabolism, and disease status.^14^, ^15^, ^16^ In this study, we found no significant association between vitamin D insufficiency or deficiency and disease duration, season of visit, latitude of residence, lipid levels, or underlying disease status in patients with BVP. It can be difficult to determine the exact cause of decreased vitamin D levels, which may also be influenced by small sample sizes and primary diseases. Additionally, owing to the retrospective design, several other potential confounders ‒ including BMI, renal function, dietary intake, vitamin D supplementation, and physical activity ‒ were either incompletely recorded or could not be reliably quantified, and therefore could not be included in the analysis.

Vitamin D is involved in the pathophysiological processes of many diseases, most notably in calcium and phosphorus metabolism, as well as bone health. However, several clinical trials and meta-analyses have investigated the association between vitamin D deficiency and type 2 diabetes, cardiovascular disease, neurological disorders, and even COVID-19, as well as the underlying mechanisms. Similarly, higher levels of vitamin D help reduce the risk of respiratory infections. This may be because vitamin D controls the innate immunity in macrophages and regulates adaptive immune responses in B-cells, T-helper cells, and T-regulatory cells.^7^^,^^17^^,^^18^ Additionally, vitamin D receptors and hydroxylases are widely expressed in the brain. Vitamin D may exert its neuroprotective effects by regulating neurotrophic factors, counteracting cytotoxicity and reducing oxidative stress. Vitamin D deficiency is a risk factor for diabetic peripheral neuropathy and is associated with the occurrence and severity of sensory polyneuropathy.^8^^,^^19^

Insufficient or deficient vitamin D levels are associated with certain vestibular system disorders. Vitamin D influences calcium metabolism and homeostasis in the vestibular endolymph. Low levels of vitamin D can promote otoconia formation and hinder the dissolution of detached otoconia. An increased incidence and recurrence rate of BPPV can be attributed to this.^11^^,^^12^^,^^20^, ^21^, ^22^ Case-control studies have shown that patients with VN have significantly lower vitamin D levels than do those in the control group. Vitamin D deficiency has been reported as a factor associated with VN, and it has been hypothesized that this association may be related to vitamin D's role in regulating inflammation and immune responses.^13^ Furthermore, the role of vitamin D in adaptive immunity and the regulation of pro-inflammatory mediator expression is a key focus of many researchers studying the autoimmune mechanisms of Meniere's disease.^10^

A meta-analysis has shown that vitamin D deficiency is common among Asians.^5^ Consistent with this finding, only 12.86% of the healthy controls in this study had normal 25(OH)D levels, and the average levels were further reduced in the BVP group. Logistic regression analysis indicated that sufficient 25(OH)D levels were independently associated with lower odds of BVP. However, the etiology of BVP is complex. No association was found between 25(OH)D levels and, serum calcium concentration, inflammatory markers, or underlying diseases, providing no clear evidence that vitamin D contributes to BVP through calcium-phosphorus metabolism or inflammatory pathways. Based on previous evidence, we hypothesize that vitamin D insufficiency or deficiency may reduce the inhibitory effect of BVP on innate immunity and the regulatory effect on adaptive immunity, thereby weakening neuroprotection and resistance to cytotoxicity. It is also important to consider reverse causation: postural instability and fear of falling in BVP may reduce outdoor activity and sunlight exposure, thereby lowering vitamin D levels independently of any pathogenic role. The case-control design cannot determine the temporal relationship between vitamin D status and BVP. Further analysis revealed no significant correlation between 25(OH)D levels and the degree of VOR impairment. However, the wide confidence intervals (Table 3) and small sample size preclude firm conclusions, and modest associations cannot be excluded. Given the exploratory nature of the subgroup and correlation analyses, no formal adjustment for multiple comparisons was applied. However, as all these analyses yielded non-significant results, the potential inflation of type I error is unlikely to have affected our overall conclusions. These findings should therefore be interpreted as exploratory.

Not all researchers believe that vitamin D levels are definitively related to the diseases under study, and most clinical studies investigating the efficacy of vitamin D supplementation in primary diseases have not yielded positive results.^23^, ^24^, ^25^ Therefore, vitamin D supplementation is not recommended as a routine treatment.^26^^,^^27^ In a study on postmenopausal women in Brazil, researchers found that vitamin D supplementation reduced the incidence of falls and improved postural balance.^28^ Another meta-analysis showed that vitamin D supplementation reduces the risk of falls in older individuals with vitamin D deficiency.^29^ Although the mechanisms by which vitamin D supplementation provides these benefits are unclear, given that postural imbalance, gait instability, and falls are the core symptoms of BVP,^1^^,^^30^ prospective studies on vitamin D supplementation in patients with BVP are clinically relevant.

## Conclusion

In summary, vitamin D insufficiency or deficiency is common in patients with BVP and may be a factor associated with this condition. However, the present case-control study cannot establish causality, and the specific mechanisms remain unclear. Reduced physical activity and insufficient sunlight exposure resulting from vestibular dysfunction may also contribute to low vitamin D levels, representing a plausible reverse causation pathway that should be considered. Further validation may be achieved by expanding the sample size of patients with BVP or conducting clinical studies on vitamin D supplementation. Relevant animal experiments are worth pursuing.

## Declaration of generative AI in scientific writing

None.

## Data availability

Data supporting the findings of this study are available from the corresponding author upon reasonable request.

## Ethical approval

This study was performed in line with the principles of the Declaration of Helsinki, and approved by the Ethics Committee of the First Affiliated Hospital of Sun Yat-sen University (Approval No [2023]179).

## Funding

This study was supported by grants from 10.13039/501100004000Guangzhou Science and Technology Program projects (2023A04J2199), Guangdong Basic and Applied Basic Research Foundation (2023A1515010491), 10.13039/501100001809National Natural Science Foundation of China (82271410), Guangzhou Basic and Applied Basic Research Foundation (2024A04J4565).

## Conflicts of interest

The authors declare no conflicts of interest.
